# High gain antipodal meander line antenna for point-to-point WLAN/WiMAX applications

**DOI:** 10.1038/s41598-024-54994-x

**Published:** 2024-03-08

**Authors:** Mohamed M. Gad, Mai O. Sallam

**Affiliations:** Nanoelectronics Integrated Systems Center, Nile University, Giza, Egypt

**Keywords:** Electrical and electronic engineering, Characterization and analytical techniques, Electrical and electronic engineering, Characterization and analytical techniques

## Abstract

This paper introduces a planar antipodal meander line antenna fabricated using RO3003 substrate. The proposed antenna is designed to radiate in the end-fire direction, achieving a maximum measured gain of 10.43 dBi within its working bandwidth, which ranges from 2.24 GHz to 2.7 GHz, covering long-range WLAN/WiMAX applications. A systematic procedure is adopted in the design process to prove its tunability to cover other application requirements in terms of gain and bandwidth. The proposed design steps show that the bandwidth and the gain can independently be controlled by adjusting specific design parameters such as the number of radiators and the scaling factor. The antenna is fine-tuned using sensitivity analysis and parametric study to guarantee optimum operation at the desired bandwidth. Experimental measurements of the fabricated design demonstrate a high degree of correlation with simulations conducted using CST and HFSS. In comparison to other end-fire antennas presented in the literature, the proposed design manifests its capability to provide high gain for WLAN/WiMAX nodes connectivity. The measurements show that the proposed traveling wave antenna exhibits a high radiation efficiency with maximum value of 99.8% at 2.35 GHz. The measured side lobe level is found to be below $-18$ dB and $-20$ dB at 2.45 GHz along the $E$- and $H$-planes, respectively. Apart from its excellent radiation performance, the antenna is characterized by its low profile and fabrication simplicity.

## Introduction

Nowadays, broadband wireless access with high data rate and long coverage is required as a result of the great advances in multimedia communications^1^. Thus, an exceptionally robust back-haul links are necessary to manage the substantially ongoing increase in demanded data rates. Two recognized wireless systems which can deliver wireless high-speed internet and wireless connections are WiFi and WiMAX which are commonly used and affordable^2,3^.

In order to extend the feasibility of long-range WiFi/WiMAX integration, high gain antennas with compact size are required to cover the band from 2.3 GHz to 2.7 GHz^4^ . End-fire antennas are known for their advantage of high gain and compact size ^5^. Furthermore, they offer another level of flexibility for their placement, as they are very simple to be implemented and integrated with other passive and active components on a single substrate^6^. One of the most common end-fire antennas is the “Yagi–Uda” antenna, which was invented in 1926 by Hidetsugu Yagi and Shintaro Uda^7^. However, its main drawback is the effect of the parasitic elements (directors) on the radiator’s input impedance, which limits its bandwidth. This leads to a complexity in the design due to the trade-off between the bandwidth and the gain^8^. To solve this problem, a folded dipole is utilized as the main radiator of the Yagi–Uda antenna^9^, which has an input impedance of 300 $\Omega$. The results reveal a high gain of 10.6 dBi with 10.42$\%$ fractional bandwidth covering the WLAN 2.4 GHz band. However, a tapered balun is required to feed the folded dipole through microstrip input line which increases the overall lateral size of the structure to 0.42 $\lambda _0$ $\times$ 0.0126 $\lambda _0$. With only one directive element, the Yagi–Uda antenna presented in^10^ achieves a wide bandwidth of 31.33$\%$. However its gain is limited to 6 dBi. The balun along the quarter wave transformer occupied nearly half the overall antenna design size. Another design of a Yagi–Uda antenna with tapered balun is studied in^11^. A bandwidth of only 7.67$\%$ with a peak gain of 8.17 dBi is achieved using two directive elements. To improve the bandwidth, the authors in^12^ studied the use of a magnetic dipole incorporated with a folded dipole as the driver element, with a wide bandwidth of 28.87$\%$. Additionally, a corrugated array as zero index metamaterial is used as the director, approaching 8.5 dBi, with a relatively large size of 1.27 $\lambda _0$ $\times$ 0.45 $\lambda _0$ $\times$ 0.0128 $\lambda _0$. Other Yagi–Uda antennas ^13–17^ in addition to diverse antenna designs including printed dipole array^18^, vivaldi antenna^19^, and substrate integrated waveguide (SIW) antenna^20^ are presented in the literature which also provide end-fire radiation.

In this work, a meander line antipodal antenna is proposed. The primary contribution of this work can be summarized as follows:

- *Simplicity*: Introducing a simple, and novel design for a traveling wave antenna based on a meander line antipodal structure (series dipole array). The design is carried out through a predefined systematic methodology which is presented in details.
- *Tunability*: The proposed design can easily be tuned to other frequencies by only calculating its guided wavelength, 50 Ω impedance line width, and satisfying the matching conditions.
- *Bandwidth enhancement*: Introducing the concept of scaling the lengths of the radiating dipoles constructing the meander line antenna to enhance both its impedance and gain bandwidths, aiming to improve its overall performance.
- *Modularity*: The design process is straight forward and can easily be reproduced or modified to match the system requirements. Initially, the main focus is directed towards optimizing the gain by adjusting the number of elements constructing the meander line antenna. Subsequently, bandwidth control is achieved through scaling the dipole arms and introducing parallel stubs for impedance matching. Finally, the flexibility to control the gain is achieved by varying the number of dipoles, which has a minimal impact on the impedance bandwidth when the number of dipoles reaches a certain predetermined value.
- *Compact size*: As compared to other end-fire antennas presented in literature (e.g. Yagi-Uda), the proposed design provides wider impedance bandwidth and higher gain with relatively compact size.

The paper is organized as follows: The "Proposed design procedure" section presents the structure and design steps of the purposed antenna. Second, using the sensitivity analysis, the effect of varying the antenna’s main geometrical parameters on its performance is studied in the "Parametric study" section. Third, the characterization of the fabricated prototype and the radiation mechanism of the antenna is presented and discussed in the "Results and discussion" section. Finally, a conclusion is conducted.

## Proposed design procedure

**Figure 1:**
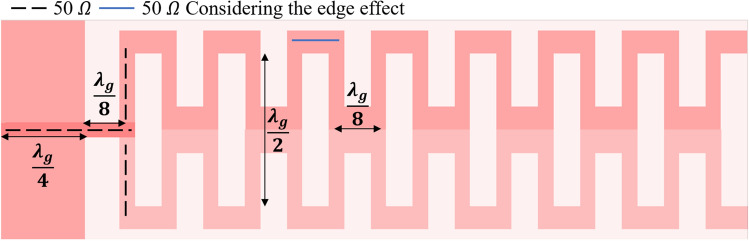
Initial design of the proposed antipodal meander line antenna.

Figure  1 shows the structure of the proposed antenna, which consists of *N* horizontal copper dipole radiators oriented along the *x*-axis, constructing the meander line. This configuration employing a traveling wave is utilized to establish a smooth transition with low reflection from a transmission line to free space. The design procedure is illustrated in a set of steps in the following subsections. First, the design of array of variable element number is studied and the contribution due the scaling effect is presented. For impedance matching between the antenna and the feeding microstrip line, two parallel stubs are placed at a calculated distance from the meander line antenna. Finally, the design parameters are fine tuned for gain and bandwidth enhancement.

### Number of elements

**Figure 2:**
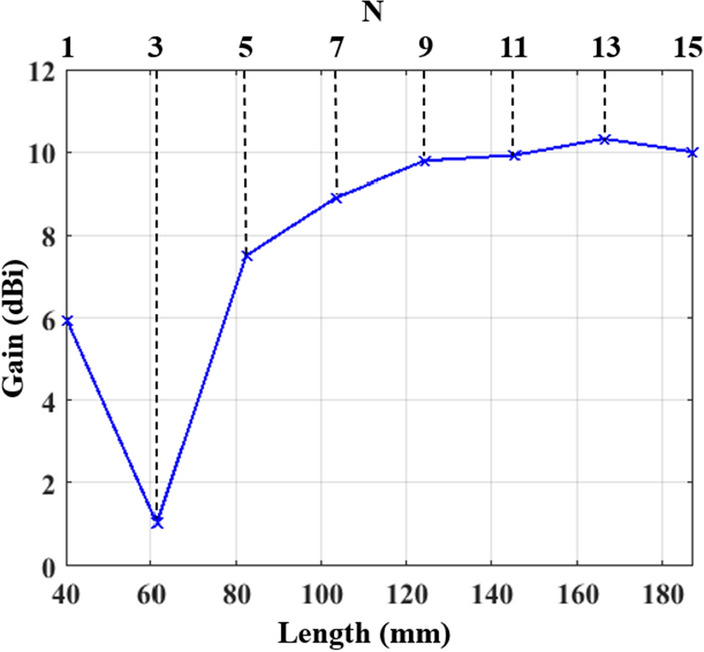
The effect of increasing the number of dipole radiators constructing the meander-line antenna on the end-fire gain.

**Figure 3:**
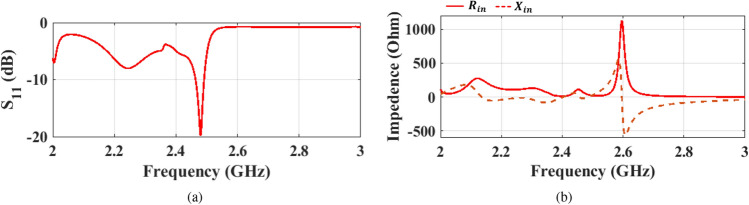
Initial meander line antenna design results: (**a**) reflection coefficient, and (**b**) input impedance.

The meander line antenna is placed on top of a Rogers RO3003 substrate ($\epsilon _{\textrm{r}}$ = 3) with a thickness of 1.527 mm. The feeding of the antenna is achieved via a 50 $\Omega$ microstrip transmission line. The backside of the substrate realizes a truncated ground plane of length “*Gnd*” connected to another mirrored meander line with the same structure and dimensions of the top meander, as shown in Figure  1. The first design step of the proposed antenna is assuming an array of simple dipole ($\lambda _g$/2) with a specified number of elements (*N*), spaced by $\lambda _g$/8. The ground plane maintains a separation of $\lambda _g$/8 from the first dipole, while its length corresponds to $\lambda _g$/4. All the radiating, feeding, and connectors are designed to have a width corresponding to 50 Ω microstrip line at the resonance frequency. To validate the proposed antenna and fulfill the desired application, the design is initially tuned at 2.45 GHz. Therefore, the widths of all dipole radiators equals 3.84 mm, corresponding to the 50 Ω impedance. However, the width of the connectors is designed to be 5.8 mm to account for the edge effecsts^21^. To optimize the antenna for higher gain while minimizing its size, a study for the end-fire gain is performed at 2.45 GHz when varying the numbers of array elements, as displayed in Figure 2. The results indicate a 6 dB gain for a single dipole element (*N* = 1), which is typical for a dipole radiator with ground reflection effects. Increasing *N* beyond 3 yields a gain boost at the expense of increased antenna size. Convergence becomes evident after *N* reaches 7, resulting in little enhancements in the end-fire gain as *N* increases and is associated with further size expansion. So there is a trade-off between achieving higher gain, when increasing the number of radiators, and obtaining a compact size antenna. The optimum design depends on the desired application and limitations on the antenna size according to the system requirements. The return loss behavior of the initial design of the meander line antenna with 7 elements is presented in Figure 3(a), revealing a narrow bandwidth due to rapid impedance variation over the WLAN/WiMAX bandwidth range.

### Bandwidth Enhancement

**Figure 4:**
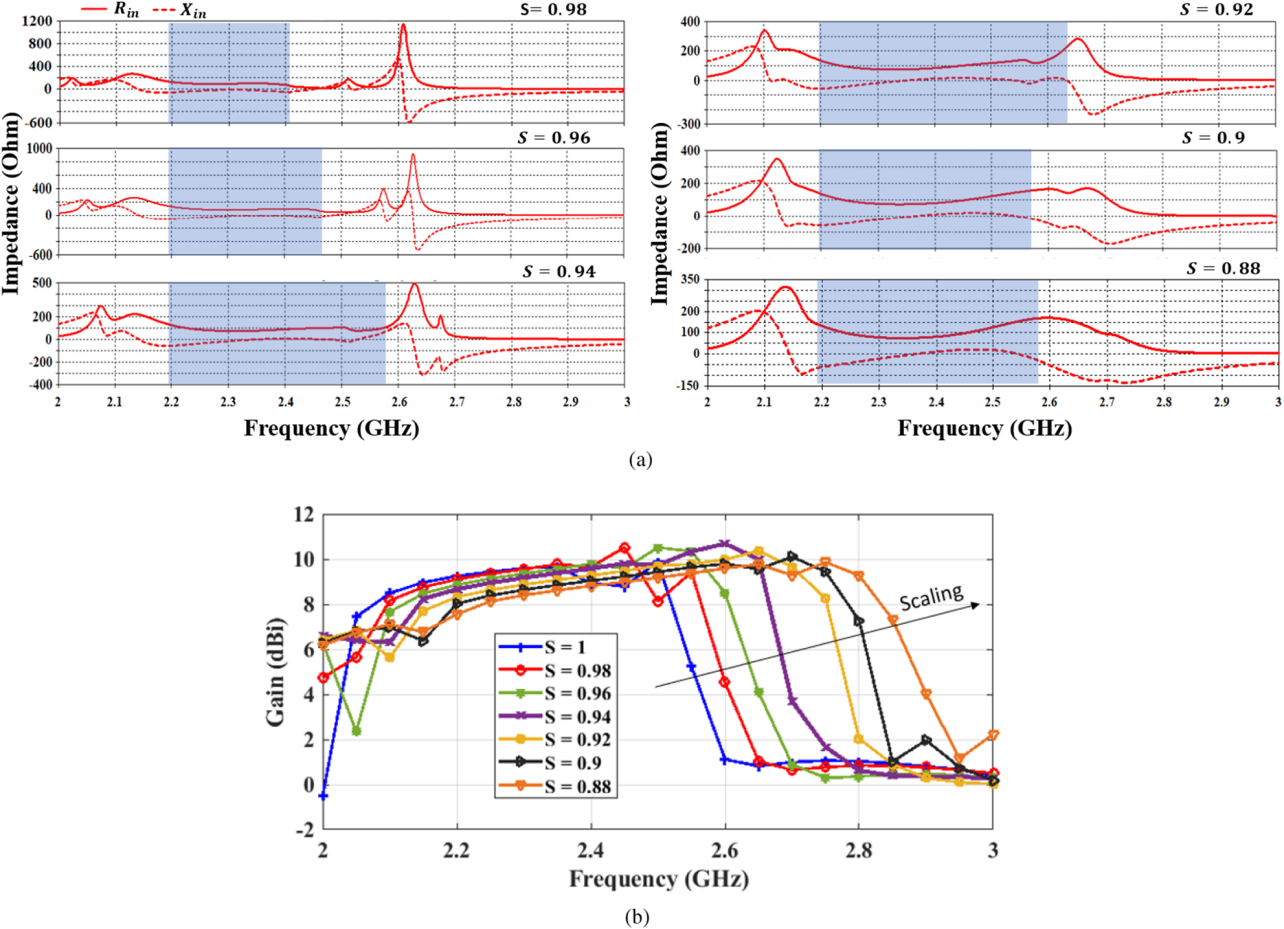
Effect of varying the scaling factor, *S*, on the antenna characteristics: (**a**) input impedance, and (**b**) end-fire gain.

**Figure 5:**
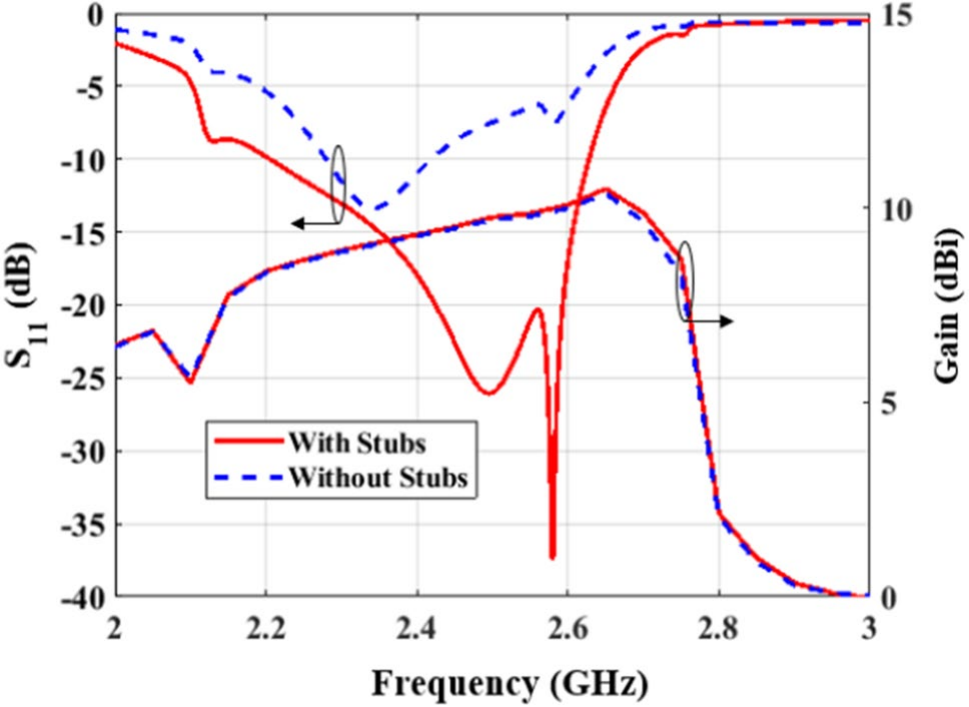
Reflection coefficient and end-fire gain of the proposed antenna with stubs and scaling.

By introducing the scaling, the meander line antenna can be viewed as an array of dipole radiators of different lengths, which are connected to each other through non-radiating connectors. The scale factor, denoted by *S*, is a constant multiplier used to relate the length of the *n*th dipole to the length of the preceding one. The discrepancy between the lengths of the dipoles leads to multi resonance modes. Therefore, the impedance bandwidth and 3–﻿dB gain bandwidth can be enhanced. Figure 4(a) shows the effect of varying the scaling factor on the input impedance of the array, where the region with semi-stable impedance behavior is highlighted. As the scaling factor deviates from unity, the resistive component of the impedance becomes more stable since the modes spreads over wider bandwidth (towards high frequencies). In fact, a reduction in *S* leads to an increased overlapping of resonance modes (i,e,. stabilizing the resulting impedance) owing to the intense variation in the dipole lengths. When *S* decreases below 0.92,the resistive component of the impedance of the higher-order modes are damped around 2.6 GHz, as shown in Figure 3(b). On the other hand, the reactive component of the impedance is strongly affected by this high-order mode, especially when *S = *0.9 or below, where the antenna tends to have a capacitive behavior. It is worth noting that, a significantly reduced scale factor may result in further improvement in the impedance stability but at the cost of the gain value at the end-fire direction. Yet, maintaining *S* at 0.92 proves to be efficient, since is leads to balancing both the gain level and bandwidth as shown in Figure 4(b). Further reduction of *S* increases the 3–﻿dB gain bandwidth at the expense of its magnitude especially at low frequencies, which is attributable to shifting the gain bandwidth to higher frequencies. At this stage, the impedance stability is targeted rather than simply achieving an input resistance of 50 Ω and a reactance of 0 Ω at a specific frequency.  This offers additional freedom in tuning the final design by using a matching network that fits a wider rage of frequencies around the central frequency of operation. For instance, the antenna can be matched by introducing two parallel stubs connected to the input feeding line. The width of the stubs has the same dimensions as the microstrip transmission line (50 Ω). By simple calculations or using the smith chart, the position and the length of the two stubs is determined to be 26.73 mm as the distance from the the first dipole and 5.04 mm as the length for a single stub. The return loss and the gain results for the proposed antenna are shown in Figure 5. The antenna covers the band from 2.2 GHz to 2.63 GHz, corresponding to a fractional bandwidth of 17.77%. Moreover, as expected, the stubs have no effect on the gain. In the next section, the antenna is fine tuned for better performance where the antenna's geometrical parameters are varied around the predetermined values to further enhance its performance.

### Design fine tuning

**Figure 6:**
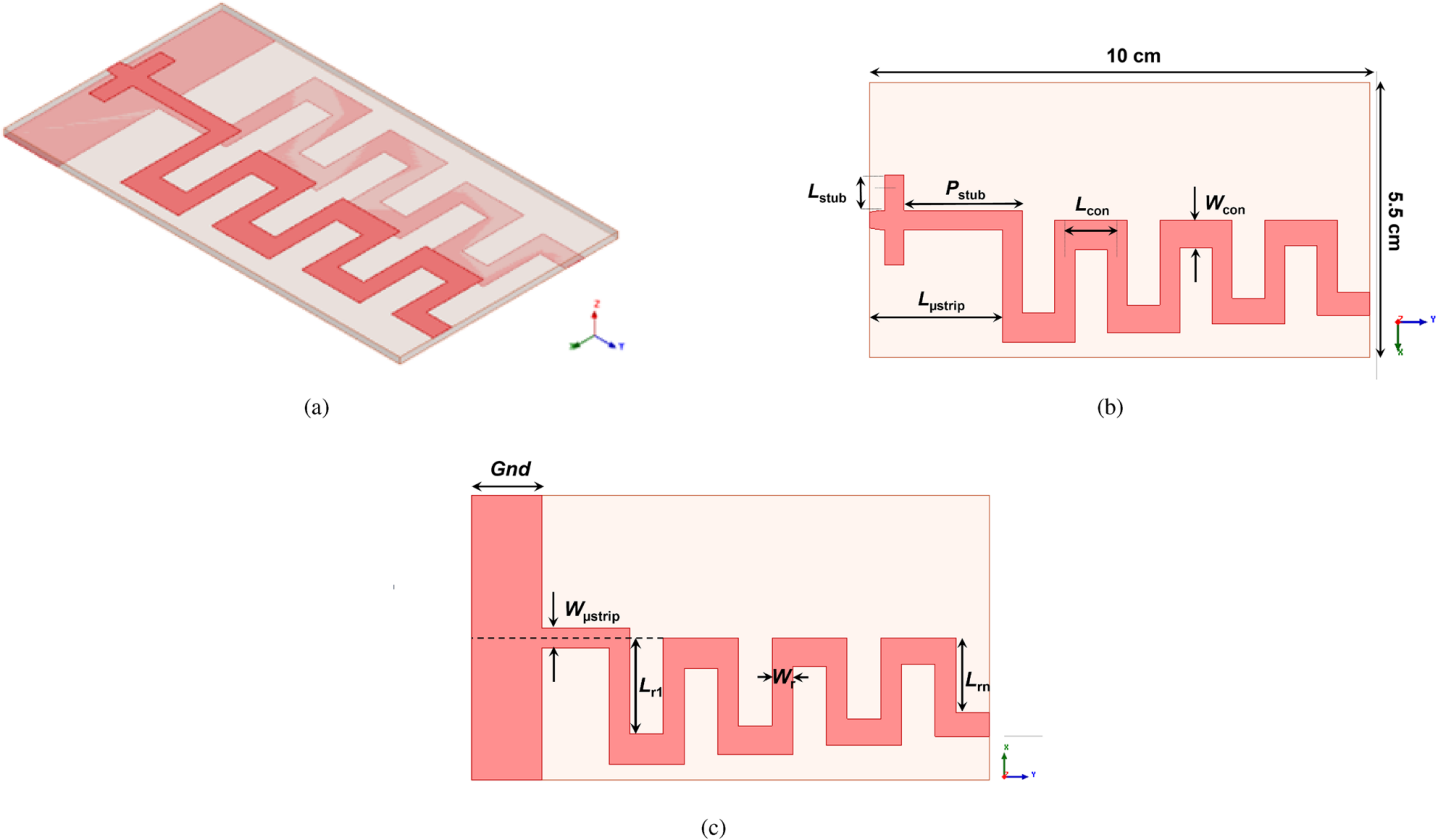
Structure of the proposed antipodal meander line antenna (**a**) 3D view, (**b**) top view, and (**c**) bottom view.

The final structure of the proposed meander line antenna is shown in Figure 6. The design of the antenna incorporates *N ﻿= 7* horizontal copper scaled dipole radiators along the *x*-axis, forming the traveling wave configuration. Each of these radiators has length $$L_{\textrm{rn}}$$ and width $$W_{\textrm{r}}$$, where the suffix “*n*” represents the order of the radiator. The dipoles are connected to each other via several connectors oriented along the *y*-axis whose lengths and widths are denoted by $$L_{\textrm{con}}$$ and $$W_{\textrm{con}}$$ respectively. The length of the $$n$$$${\text{th}}$$ dipole is related to the preceding one through the equation: $$L_{\textrm{rn}}$$ = $$S$$ $$\times$$ $$L_{\mathrm {rn-1}}$$, where *S* is the scale factor that has been introduced in the previous subsection which demonstrated that *S* significantly influences the characteristics of the antenna. The stubs have lengths $$L_{\textrm{stub}}$$ and are located at distance $$P_{\textrm{stub}}$$ from the meander line antenna. To enhance the proposed antenna, the geometrical parameters of the structure are varied within a suitable range from the initial design parameters in order to fine tune the antenna for best performance. Table 1 list the antenna’s parameters along with its initial values, lower,  and upper limits of the sweeping range, and the final optimum values. Most of the dimensions are listed in terms of guided wavelength for reference. The stub size and location  are calculated such that they satisfy the matching conditions with the feeding microstrip, as mentioned earlier. The optimal parameters for the 2.45 GHz antenna design are identified in the table after considering both the gain and bandwidth in the study performed. The final design has an end-fire gain with maximum value of 10.43 dBi within the antenna's working bandwidth, and a fractional impedance bandwidth of 19.34%. The next section shows how the antenna is fine turned with the aid of the sensitivity analysis and parametric study of the antenna's geometrical parameters.

**Table 1:** Comparison between the geometrical dimensions of the initial and final antenna designs.

Parameter	Initial dimensions	Sweep range	Proposed electrical dimensions	Final design dimensions
$L_{\text {con}}$	$\lambda _g/8$	$0.1$–$0.1\lambda _g$	$\lambda _g/8$	10.5 mm
$L_{r1}$	$\lambda _g/4$	$0.15$–$0.3\lambda _g$	$0.22\lambda _g$	18.5 mm
*Gnd*	$\lambda _g/4$	$0.15$–$0.3\lambda _g$	$0.161\lambda _g$	13.5 mm
$L_{\mu \text { strip}}$	$0.375\lambda _g$	$0.3$–$0.4\lambda _g$	$0.315\lambda _g$	26.5 mm
$W_{\text {con}}$	5.8 mm	3–7 mm	–	6 mm
$W_{\text {rad}}$	3.84 mm	3–7 mm	–	4 mm
*S*	0.92	0.88–0.98	–	0.92
$L_{\text {stub}}$	–	–	–	7.08 mm
$P_{\text {stub}}$	–	–	–	28.5 mm

**Table 2:** Sensitivity analysis of the proposed meander antenna due to $\pm 10$ $\%$ perturbation of its geometrical parameters.

Parameter	Bandwidth sensitivity		Gain sensitivity	
	GHz /+10%	GHz /−10%	GHz /+10%	GHz /−10%
$L_{\textrm{con}}$	$\hspace{0.2cm} 0.022$	$\hspace{0.2cm} 0.032$	$-0.424$	$-0.609$
$L_{\textrm{r1}}$	$-0.232$	$-0.381$	$-0.99$	$-1.899$
*Gnd*	$\hspace{0.25cm} 0.008$	$-0.008$	$-0.023$	$-0.017$
$L_{\mathrm {\mu strip}}$	$-0.474$	$-0.333$	$-1.789$	$-1.92$
*S*	$-0.328$	$-0.016$	$-0.34$	$-1.894$
$W_{\textrm{rad}}$	$-0.2$	$-0.009$	$-1.614$	$-0.08$
$W_{\textrm{con}}$	$\hspace{0.25cm} 0.031$	$-0.039$	$\hspace{0.2cm} 0.12$	$-0.122$

## Parametric study

**Figure 7:**
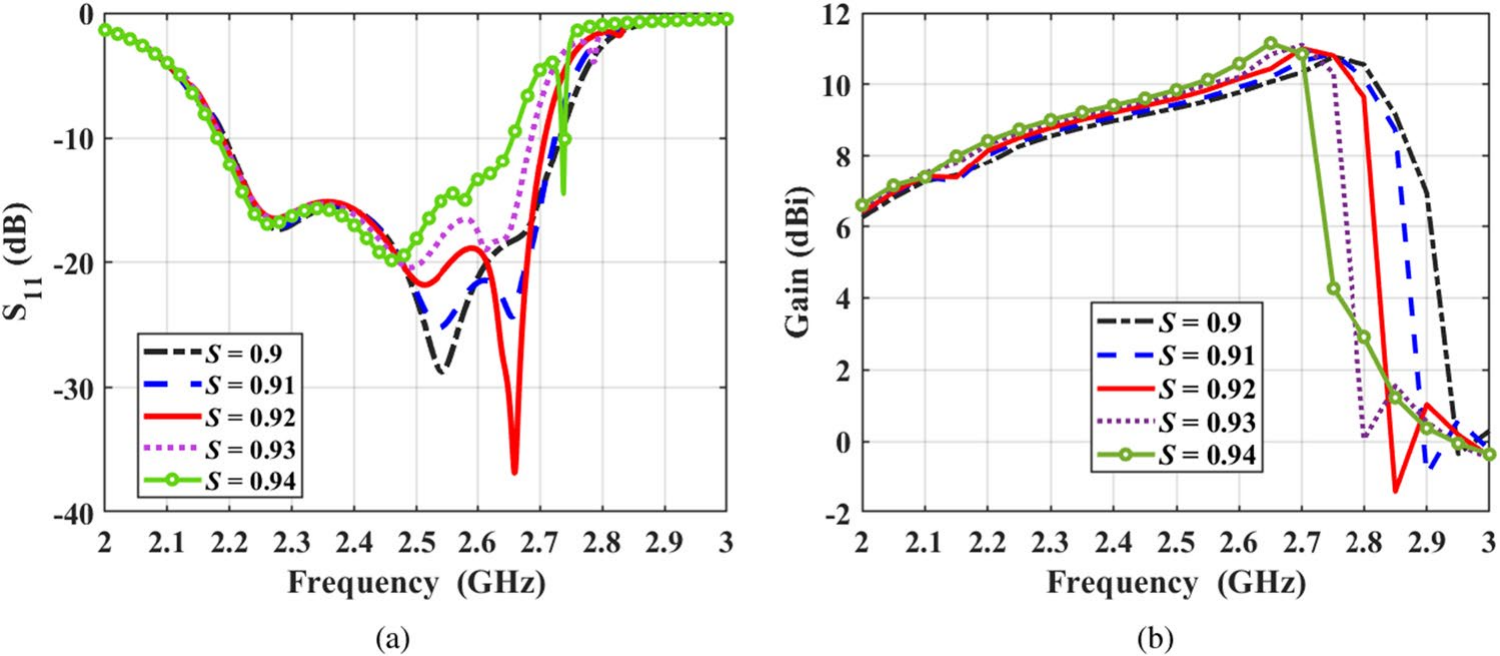
Effect of varying *S* on the (**a**) reflection coefficient, and (**b**) end-fire gain of the antenna, while keeping all other parameters at their optimum value.

**Figure 8:**
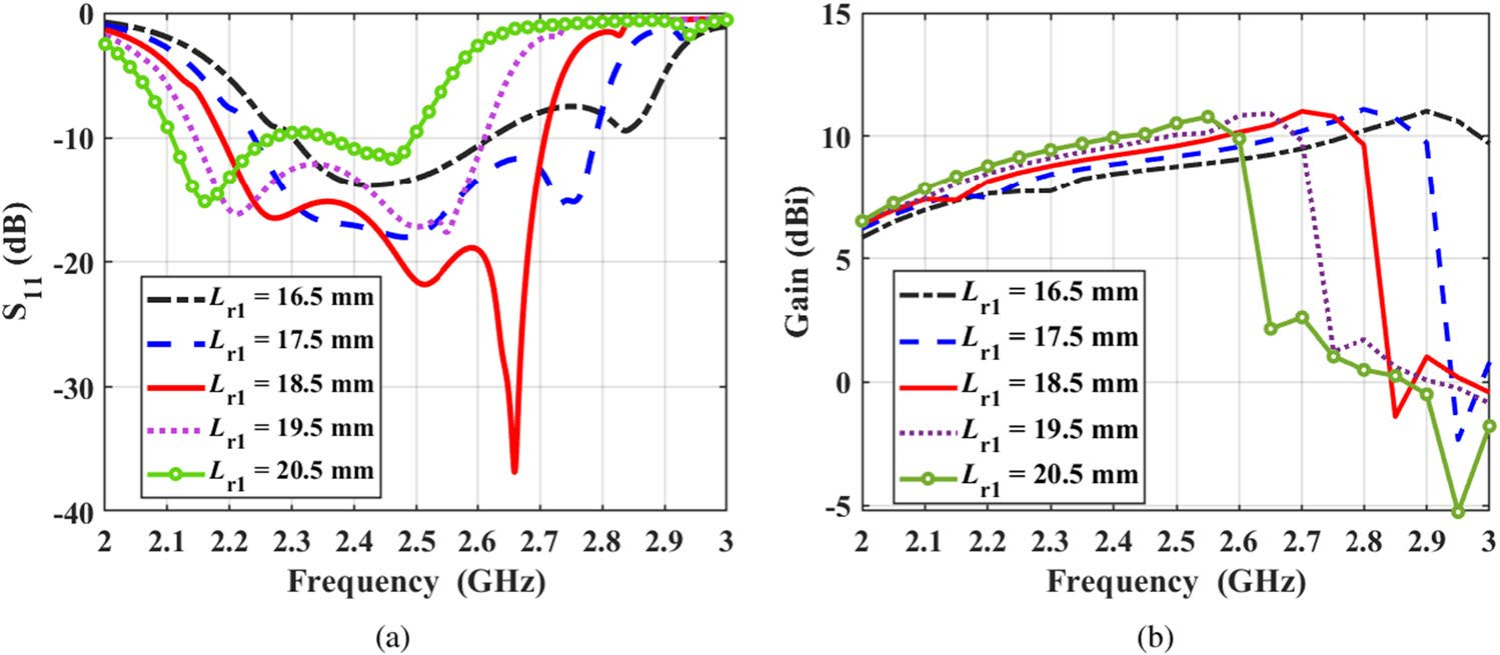
Effect of varying $L_{\textrm{r1}}$ on the (**a**) reflection coefficient, and (**b**) end-fire gain of the antenna, while keeping all other parameters at their optimum value.

The effect of varying the dimensions of the proposed antipodal meander line antenna is discussed in this section. The study is performed while keeping the length and width of the substrate fixed at 10 cm and 5.5 cm respectively. First, a sensitivity analysis is performed and presented in Table 2 to study the impedance bandwidth and the maximum end-fire gain due to $$\pm 10$$$$\%$$ variations of the antenna’s geometrical parameters. It can be concluded that the most significant parameters affecting the antenna’s performance are: $$L_{\textrm{r1}}$$, *S*, and $$L_{\mathrm {\mu strip}}$$. The parameter $$L_{\mathrm {\mu strip}}$$ is the most influential parameters that impacts both the reflection coefficient and gain. This results from altering the matching condition, which dramatically affects its impedance. Moreover, adjusting $$L_{\mathrm {\mu strip}}$$ alters the number of radiators, consequently affecting the resulting gain. Additionally, the scale factor *S* has considerable effect in both bandwidth and gain. The impedance bandwidth of the antenna increases since the dipole lengths constructing the meander line have more discrepancy, which are validated in previous section. Since the length of the first dipole element which has the largest value is not affected by the scale factor, the starting point of the bandwidth is the same for all values of *S*. Although increasing the scale factor has advantageous effect on enhancing the impedance bandwidth of the antenna, the maximum gain over this band decreases (see Figure 7). This trade-off allows the control of the antenna’s bandwidth to a certain limit until no further enhancement of the bandwidth can be obtained. Other parameters including $$W_{\textrm{con}}$$, and *Gnd* have much smaller effect on the antenna’s performance, while $$L_{\textrm{con}}$$ has a considerable effect on the radiated gain only. This can be explained from the fact that the lengths of the connectors mainly control the spacing between the dipole radiators constructing the antenna. $$L_{\textrm{con}}$$ also play a major role in determining the number of dipoles since the substrate length is kept fixed at 10 cm in this study. On the other hand, the parameter $$W_{\textrm{rad}}$$ affects the gain mainly since it controls the value of the guided wavelength; hence, it changes the current distribution along the meander line antenna.

The effect of varying $L_{\textrm{r1}}$ on the reflection coefficient and gain of the proposed antenna are shown in Figure 8. This parameter controls the lengths of all radiators which are dependent on each other through the scale factor *S*. It is obvious that as $L_{\textrm{r1}}$ increases, the impedance bandwidth of the antenna shifts to lower frequencies since the lengths of all dipole radiators increase. In addition, $L_{\textrm{r1}}$ strongly affect the matching level of the antenna, which is optimum at $L_{\textrm{r1}}$ = 18.5 mm. It is also clear that decreasing the lengths of the radiators leads to a more stable gain over the working bandwidth of the antenna at the cost of the matching level.

**Figure 9:**
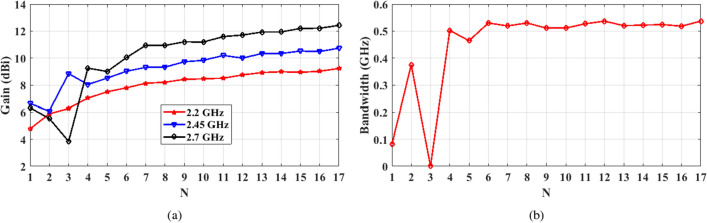
Effect of increasing the number of dipoles constructing the meander line antenna on its (**a**) end-fire gain, and (**b**) impedance bandwidth.

Figure 9(a) shows variation of the end-fire gain in response to increasing the number of arms constructing the meander line antenna. In this case, the length of the substrate is variable according to the number of dipole arms. For *N *= 1, the length of the meander line corresponds to $\lambda _g$/2 approximately at the middle of the bandwidth (i.e. 2.45 GHz). When the number of turns increases the radiation pattern is strongly affected by the total length of the meander line as it controls the direction and strength of the current along each arm of the meander line causing constructive interference at specific directions and destructive interference at other directions. Thus, the gain along the end-fire direction is very sensitive in the range between $N=1$ until $N=7$. Further increase of the number of turns (*N*>7) enhances the end-fire directivity; however, it can be noticed that the enhancement becomes less effective as the number of dipole arms increases. In this case, the antenna radiates a travelling wave along the end-fire direction. It is worth noting that when the total length of the meander line becomes multiples of $\lambda _g$, its impedance bandwidth becomes almost unchanged, as shown in Figure 9(b) since it can be seen as a long transmission line.

## Results and discussion

**Figure 10:**
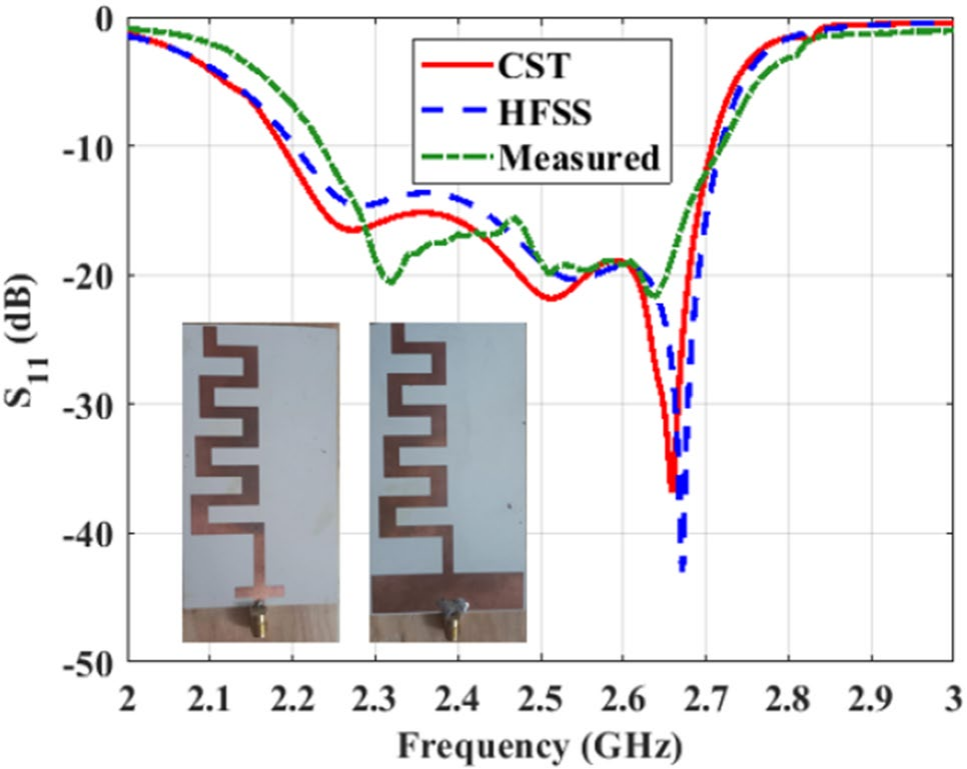
Reflection coefficient of the antipodal meander line antenna versus frequency.

**Figure 11:**
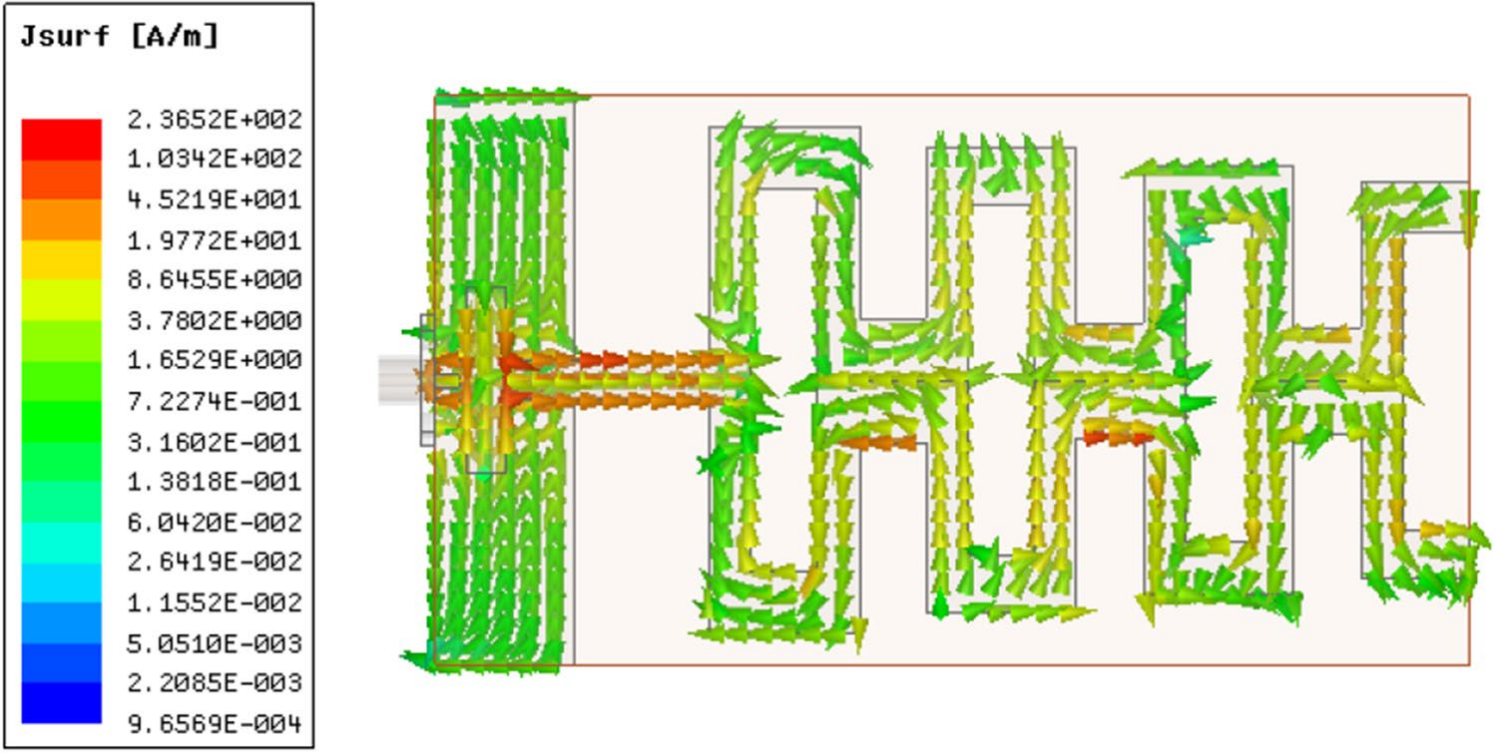
Current distribution along the top and bottom sides of the antipodal meander line antenna at 2.7 GHz.

**Figure 12:**
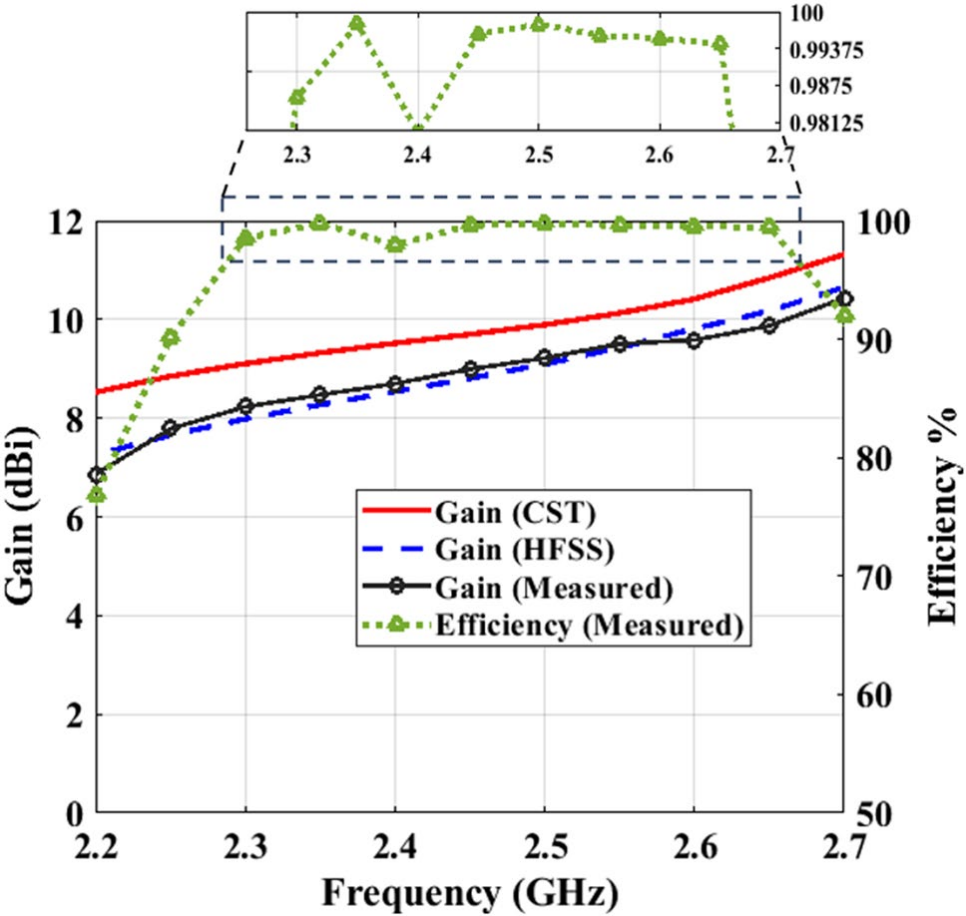
End-fire gain and radiation efficiency of the antipodal meander line antenna versus frequency.

**Figure 13:**
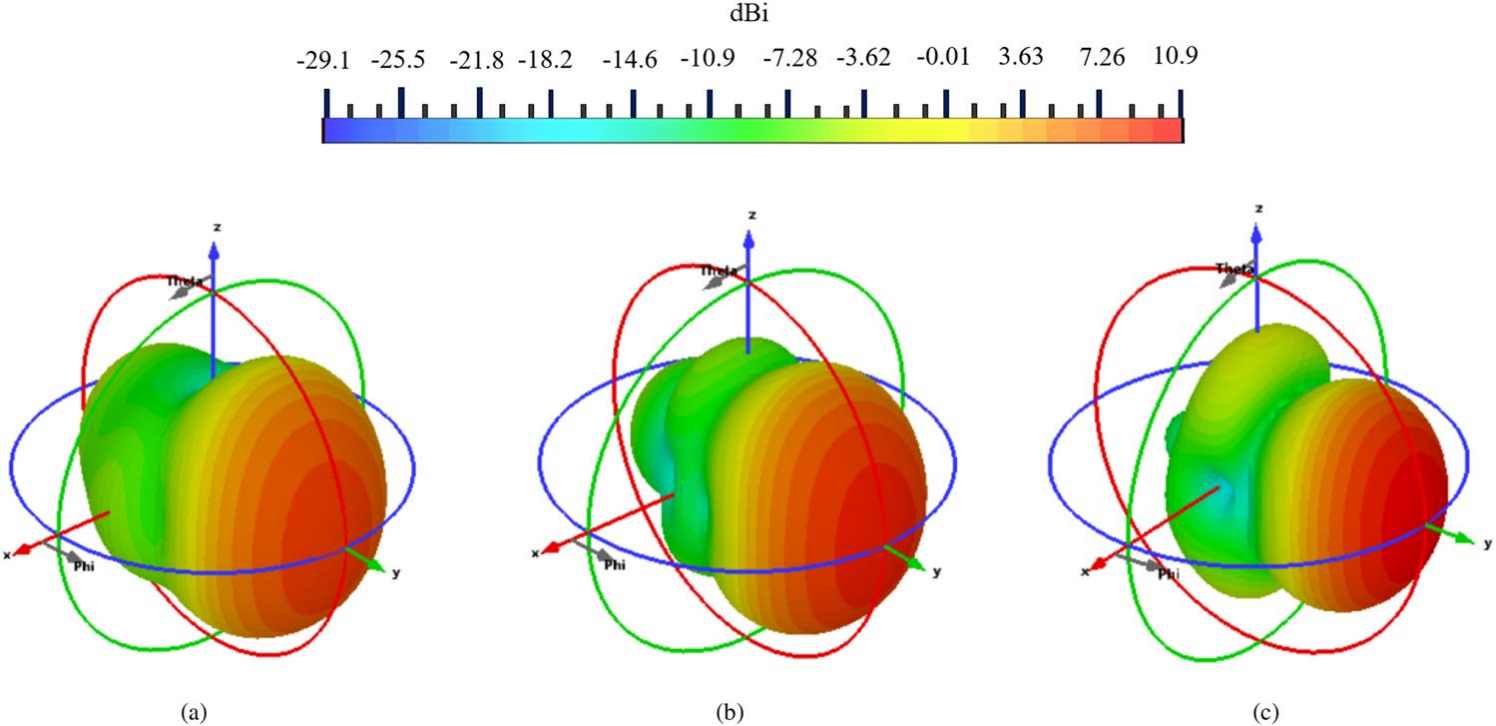
3D radiation radiation pattern of the proposed antipodal meander line antenna at (**a**) 2.2 GHz, (**b**) 2.45 GHz and (**c**) 2.7 GHz.

This section presents the characteristics of the proposed antipodal meander line antenna. Figure 10 illustrates a comparison between the simulated and measured reflection coefficient of the antenna. Both CST and HFSS are used to verify the results before fabrication, taking into account the presence of the SMA connector. The results reveal very good agreement between CST, HFSS and measurements, where multi-resonance occurs with only slight differences in the working bandwidth of the antenna. Simulations (measurements) showed that the antenna bandwidth ranges from 2.2 GHz– 2.7 GHz (2.24 GHz– 2.7 GHz), which is suitable for WiFi and WiMAX applications. To clarify the radiation mechanism of the proposed structure, Figure 11 shows the surface current along the top and bottom sides of the antenna at 2.7 GHz. It is clear that the currents along the 1st, 3rd, and 4th dipoles, which we refer to as Group 1, are in-phase. Meanwhile, currents along the 2nd, 5th, 6th, and 7th dipoles are also in-phase but they have 180^∘^ phase difference with Group 1. Thus, the proposed structure can be approximated as an array of seven dipoles with an array factor expressed as: A.F. = $\sum _{i=1}^{7} \pm W_{i} e^{-jnkd}$, where the + sign is assigned for the dipoles of Group 1, while the −ve sign refers to the other dipoles, $W_{\textrm{i}}$ is the weight of the current of each dipole, *k* is the propagation constant, and *d* is the separation between each two horizontal dipoles, which is $\lambda _g$/8 approximately at 2.7 GHz. Figure 12 shows the end-fire gain and radiation efficiency versus frequency. The results demonstrate a consistent gain behaviour between measurements and simulations. Clearly, perfect matching is observed with HFSS. The maximum gain of the antenna occurs at 2.7 GHz with a value of 10.43 dBi (10.65 dBi) according to measurements (HFSS). The figure also demonstrates that the antenna has high radiation efficiency that nearly approaches 100$\%$ in most of its working bandwidth, with a maximum value of 99.8$\%$ at 2.35 GHz. The highest remarkable radiation efficiency is due to the low loss tangent of Rogers 3003, and also reveals good optimization and tuning of the proposed antenna. The earlier study presented in^22^ achieved a peak radiation efficiency of 98.75% with the utilization of a Rogers substrate. Similarly, the proposed design in^23^ demonstrated a notable radiation efficiency, reaching as high as 99.1% also employing a Rogers substrate.

**Figure 14:**
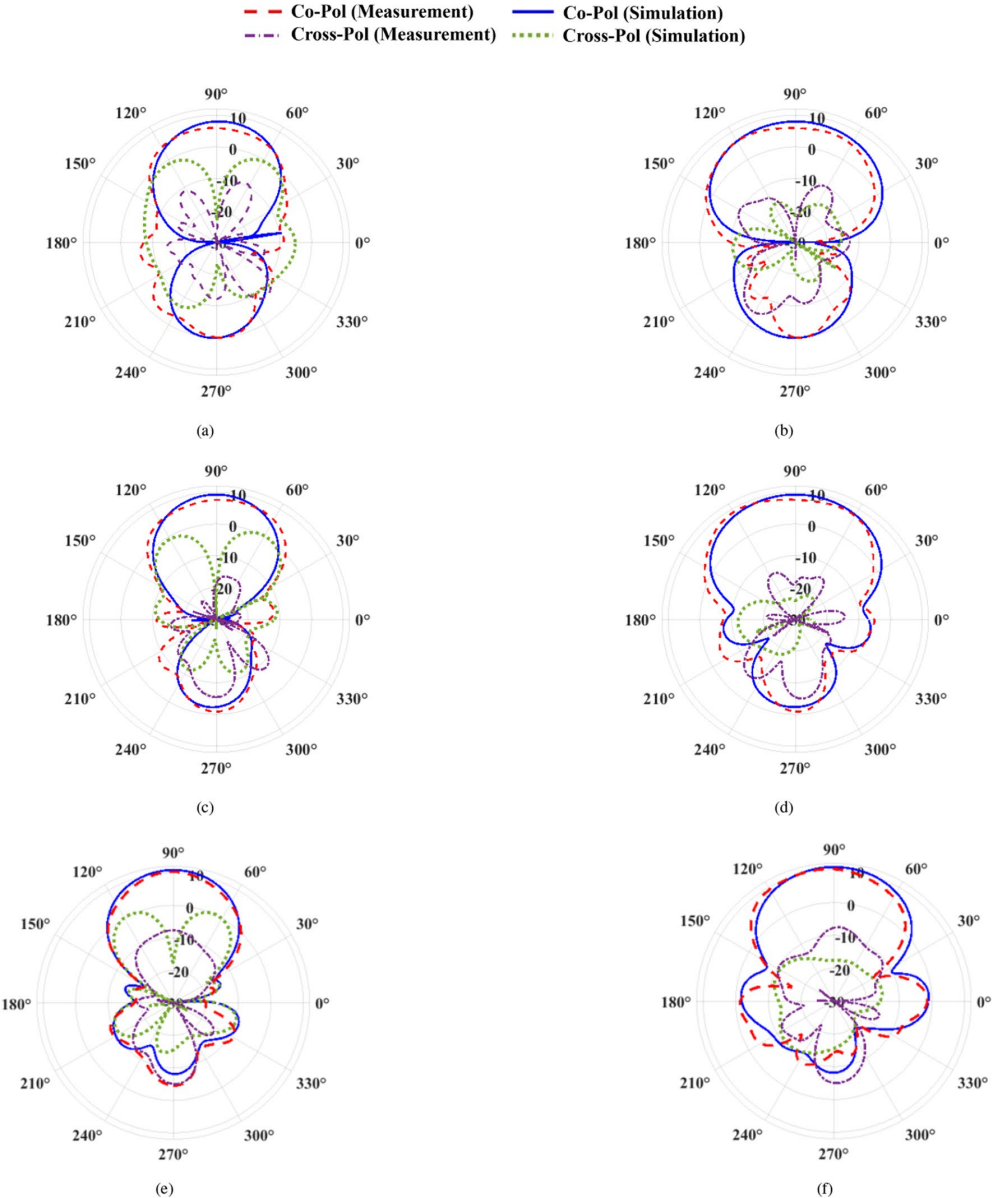
2D radiation pattern of the proposed antipodal meander line antenna at (**a**) 2.2 GHz ($E$-plane), (**b**) 2.2 GHz ($H$-plane), (**c**) 2.45 GHz ($E$-plane), (**d**) 2.45 GHz ($H$-plane), (**e**) 2.7 GHz($E$-plane), and (**f**) 2.7 GHz ($H$-plane).

**Figure 15:**
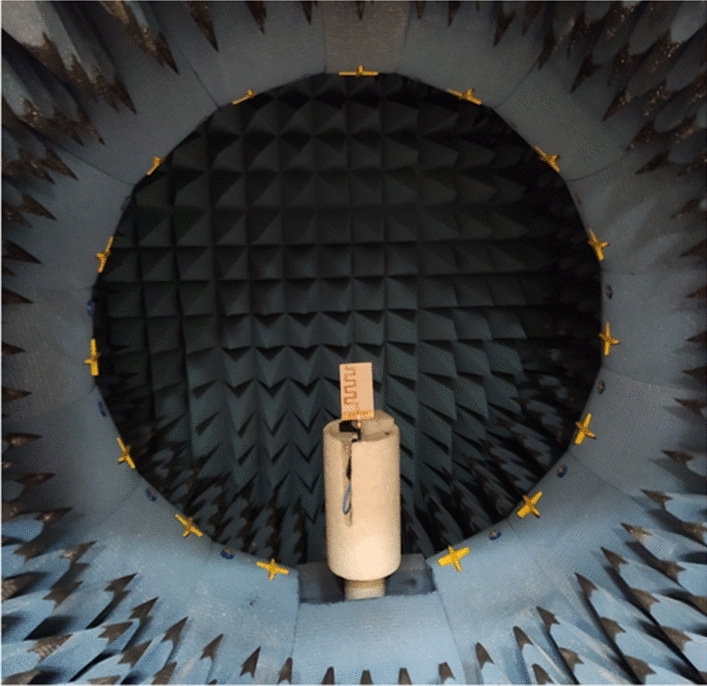
Setup of the measurement of the antenna radiation characteristics.

The 3D radiation pattern of the proposed antenna is shown in Figure 13 as simulated using CST at 2.2 GHz, 2.45 GHz, and 2.7 GHz representing the start, middle, and end point of the working bandwidth of the antenna. The radiation of the antenna is along the end-fire direction (+ve *y*-axis) with maximum directivity (radiation efficiency) of 8.13 dBi (99.2$\%$), 9.31 dBi (99.6$\%$), and 10.9 dBi (98.6$\%$ ), respectively. The 2D radiation patterns of the antenna at 2.2 GHz, 2.45 GHz, and 2.7 GHz are shown in Figure 14 for both the $E$-, and $H$-planes. Excellent matching between simulations and measurements is observed. The Half Power Beam Width (HPBW) of the antenna for both its $E$- and $H$-planes, respectively, is $51.9^\circ$, and $89^\circ$ at 2.2 GHz, $49^\circ$, and $82.4^\circ$ at 2.45 GHz, and $43.3^\circ$, and $62.6^\circ$ at 2.7 GHz. The figure also shows that the side lobe levels are below $-11.5$ dB, and $-19$ dB at 2.2 GHz along the $E$-, and $H$-planes, respectively. Furthermore, these levels are observed to be below $-18$ dB and $-12$ dB at 2.45 GHz, and below $-25$ dB and $-10$ dB at 2.7 GHz. The antenna also shows high polarization purity where the cross-polarization levels along the end-fire direction are 33.5 dB at 2.2 GHz, 26.26 dB at 2.45 GHz, and 17.97 dB, at 2.7 GHz. The measurement setup for calculating the radiation characteristics of the proposed antenna is shown in Figure 15.

Table 3 illustrates a comparison between our proposed structure and other designs presented in the literature in terms of the impedance bandwidth, gain, and electrical size at the resonance frequency. Compared to^9^, and^15^, our proposed antenna has wider bandwidth, smaller size, and comparable gain. Other structures presented in the literature provide wider impedance bandwidths such as^10,12^ with smaller gain and larger size. Meanwhile, the proposed designs in^11,13,14^ provide smaller fractional bandwidth and gain with a comparable size to our proposed design. To minimize the design size at the cost of the resulting gain , authors in^16,17^, and^24^ achieve a fractional bandwidth of 15.04%, 46.5%, and 22%. The mitigation of high gain requirements diminishes the complexities and allows for greater flexibility in achieving higher impedance matching, as the case in^25,26^. To achieve higher gain, larger size is required as the case in^27^, where a size of 11.33 $\lambda _0$ $\times$ 11.33 $\lambda _0$ $\times$ 0.1744 $\lambda _0$ is required to get a 20 dBi gain with fractional bandwidth of 10%. In comparison to the design outlined in^18^, our approach delivers a greater bandwidth and gain within a reduced size. We also proposed other versions of the antenna with smaller size The design proposed in^28^ introduces a more complex two-layer configuration, resulting in a notable bandwidth enhancement of up to 34.19%. However, it is important to note that the fabrication of such designs is marked by complexities related to layer alignment and antenna positioning. In practical commercial applications, a preference is often given to single-layer antennas despite the potential for substantial enhancements achievable through the use of multiple layers, as obtained by metasurface antennas. The simplicity and feasibility of one-layer antennas align with practical considerations in commercial settings. The suggested planner single layer meander line antenna provides flexibility in controlling the bandwidth and radiation characteristics by selecting appropriate geometrical antenna parameters such as scale factor or number of elements that can provide wider impedance bandwidth or higher gain.

## Conclusion

A new antipodal meander line antenna for WiFi/WiMax applications is presented in this paper. A step-by-step design procedure is showcased, illustrating the scalability and isolation of the proposed design. A parametric study is conducted to investigate the impact of varying the antenna’s geometrical parameters on its impedance bandwidth and radiation characteristics. Subsequently, the antenna is fine-tuned for best performance. The final design dimensions are presented in terms of guided wavelength for standardization. The antenna is then fabricated and characterized, demonstrating a strong agreement between simulations and measurements. The optimal antenna is distinguished by its wide bandwidth of 19.35%, accompanied by a maximum end-fire gain of 10.43 dBi at 2.7 GHz. The operational bandwidth encompasses the WiFi band (2.4 GHz–﻿2.48 GHz), WiMAX rel 1 (2.3 GHz–﻿2.4 GHz), and WiMAX rel 1.5 (2.5 GHz–﻿2.69 GHz), exhibiting high efficiency and low side lobe levels. Compared to other end-fire antennas presented in the literature, the proposed design demonstrates high impedance bandwidth, directional radiation pattern, compact size, and fabrication simplicity.

**Table 3:** Comparison between our proposed meander line antenna with diverse antennas published in the literature.

Refs.	Bandwidth (%)	Gain (dBi)	Electrical Size
^9^	10.42	10.6	1.16$\lambda _0$ $\times$0.42 $\lambda _0$ $\times$ 0.0126 $\lambda _0$
^10^	31.44	6	0.84 $\lambda _0$ $\times$0.64 $\lambda _0$ $\times$ 0.0125 $\lambda _0$
^11^	7.67	8.167	0.82 $\lambda _0$ $\times$0.8 $\lambda _0$ $\times$ 0.005 $\lambda _0$
^12^	28.87	8.5	1.27 $\lambda _0$ $\times$ 0.45 $\lambda _0$ $\times$0.0128 $\lambda _0$
^13^	16.12	8.36	1.88 $\lambda _0$ $\times$0.18 $\lambda _0$ $\times$0.006 $\lambda _0$
^14^	13.5	8.62	1.52 $\lambda _0$ $\times$ 0.2$\lambda _0$ $\times$ 0.012 $\lambda _0$
^15^	3.61	10.61	1.31 $\lambda _0$ $\times$1.31 $\lambda _0$ $\times$ 0.004 $\lambda _0$
^16^	15.04	4.8	0.55 $\lambda _0$ $\times$0.47 $\lambda _0$ $\times$ 0.0128 $\lambda _0$
^17^	46.5	6	0.52 $\lambda _0$ $\times$0.22 $\lambda _0$ $\times$ 0.009 $\lambda _0$
^18^	13.75	9.6	1.44 $\lambda _0$ $\times$0.48 $\lambda _0$ $\times$ 0.108 $\lambda _0$
^24^	22	6	0.96 $\lambda _0$ $\times$ 0.8 $\lambda _0$ $\times$ 0.174 $\lambda _0$
^28^	34.19	8.39	0.5625 $\lambda _0$ $\times$ 0.49 $\lambda _0$ $\times$ 0.027 $\lambda _0$
^27^	10	20	11.33 $\lambda _0$ $\times$ 11.33 $\lambda _0$ $\times$ 0.1744 $\lambda _0$
^25^	153.22	5	0.25 $\lambda _0$ $\times$ 0.17 $\lambda _0$ $\times$ 0.06 $\lambda _0$
^26^	107.35	4.91	0.33 $\lambda _0$ $\times$ 0.57 $\lambda _0$ $\times$ 0.027 $\lambda _0$
This work (*N* = 2)	15.29	6.08	0.39 $\lambda _0$ $\times$0.45 $\lambda _0$ $\times$ 0.0125 $\lambda _0$
This work (*N* = 4)	20.54	8.99	0.56 $\lambda _0$ $\times$0.45 $\lambda _0$ $\times$ 0.0125 $\lambda _0$
This work (*N* = 7)	19.35	10.43	0.82 $\lambda _0$ $\times$0.45 $\lambda _0$ $\times$ 0.0125 $\lambda _0$

## Data Availability

All data generated or analysed during this study are included in this published article.
